# A Novel, Sport-Specific EMG-Based Method to Evaluate Movement Efficiency in Karate Punching

**DOI:** 10.3390/sports13070218

**Published:** 2025-07-07

**Authors:** László Csákvári, Bence Kopper, Tamás Horváth

**Affiliations:** 1 Department of Combat Sports, Hungarian University of Sports Science, 1123 Budapest, Hungary; csakvari.laszlo@tf.hu; 2 Department of Kinesiology, Hungarian University of Sports Science, 1123 Budapest, Hungary; kopper.bence@tf.hu; 3 Research Center for Sports Physiology, Hungarian University of Sports Science, 1123 Budapest, Hungary

**Keywords:** kinematics, electromyography, acceleration, karate, reverse punch

## Abstract

Background: This study aimed to develop a method to analyze the kinetic and kinematic characteristics of the traditional karate Gyaku Tsuki (reverse punch), focusing on the activation sequence of lower and upper extremities and trunk muscles during execution. Methods: An elite male (N = 1) karate athlete (in kata) performed 20 Gyaku Tsuki punches while equipped with 16 wireless surface EMG sensors integrated with 3-axis accelerometers. The five punches with the highest forearm acceleration were selected for analysis. EMG, accelerometer, and synchronized video data were recorded and processed. Results: A novel visualization technique was developed to represent muscle activation over time, distinguishing a spectrum of 0–25–50–75–100% activation levels. Muscle activation times for arm, leg, and trunk muscles ranged from −0.31 to −0.11 s relative to punch execution, indicating rapid, coordinated muscle engagement. Conclusions: This method enables detailed analysis of muscle activation patterns in karate punches. It offers valuable insights for biomechanics researchers and practical applications for coaches aiming to enhance performance and prevent injuries through better understanding of movement dynamics.

## 1. Introduction

Biomechanical analysis of sports movements such as gait, running, and cycling is well-established. Three-dimensional motion capture systems, force plates, and EMG are some of the most frequently used devices, with which universally accepted measurement protocols are performed [1,2,3,4]. However, in martial arts research, data collection [5] and processing are not as straightforward as in gait or running analysis. Martial arts movements are not as restricted, like, for example, the circular cycling motion, therefore, the individual execution pattern of a movement varies significantly between individuals [6]. Determination and classification of a martial arts movement through biomechanical variables is difficult even with objective measurements [7]. The comparison of executions and determination of good performance is highly subjective without universally accepted measurement methodology. The most significant problem is that as the movement pattern is not pre-determined, therefore, the collection of different variables of biomechanical movement data and the consequent determination of average values to be used as a baseline for comparison is very difficult [8].

Traditional karate is special compared to other martial art forms, because the optimal movement executions are pre-determined through the kata system [9]. Therefore, it would be expected that a usable biomechanical analysis methodology would have already been developed for karate. But, according to our knowledge, no such methodology has been introduced until now. Consequently, the aim of this paper is to present a novel method enabling objective analysis and classification of karate techniques.

**Figure 1:**
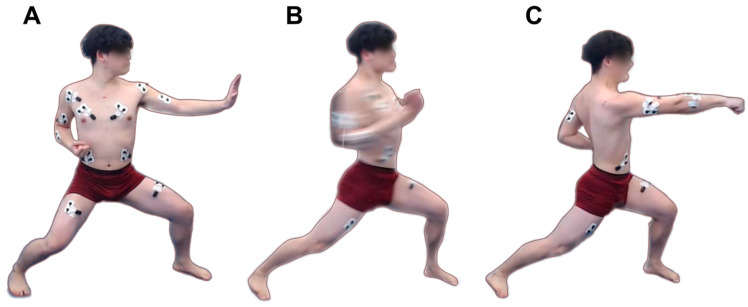
Initial (**A**), middle (**B**), and terminal (**C**) phases of a correctly executed Gyaku Tsuki. Figure displays the positioning of surface EMG/Accelerometer sensor units on the athlete.

To present the system step-by-step we have chosen the Gyaku Tsuki punch, which is one of the most frequented commonly used karate techniques in traditional karate and free style sparring [10]. The Gyaku Tsuki is a punch delivered with the opposite hand of the front leg [11]. A strong, stable stance [12] and a straight trunk perpendicular to the floor is necessary for the execution of this punch [13]. The stance makes stable connection with the ground; the perpendicular trunk posture helps to absorb the reaction force of the target and keeps the balance of the individual in the moment of impact [14]. The execution of a good Gyaku Tsuki needs the rotation of the pelvis, because pelvic rotation introduces additional momentum to the trunk, while the hips remain on the same height [15]. The punch may lose effectiveness if the center of gravity is not pushed forward parallel to the direction of the attack. Punch execution phases are depicted in Figure 1.

The punch starts with the rotation of the pelvis, which transmits power to the trunk, shoulder, arm, and fist and culminates in a strong impact force on the target. This impact can be achieved with the maximum tension of all muscles involved in the motion. At the end of the execution, the body reaches the target as a one-solid mass [16].

The delivered impact is further augmented by the reverse motion of opposite arm (hikite). At the end of the movement the punching arm, the backward pulled opposite arm, and the pelvic rotation is stopped simultaneously [14,15,16].

## 2. Materials and Methods

The test subject is an elite male karate contestant (male, age: 21 years, height: 168 cm, weight: 66 kg), who holds a third dan black belt and is ranked among the top eight in the All-Japan Championship. The subject has been practicing karate for 18 years, trains regularly and was in good physical condition with no injuries to report at the time of measurement.

The test subject was instrumented with 16 (8-8 on each side) wireless surface EMG (sEMG) sensor units (MiniWave, Cometa Italy, Milan, Italy), which also comprised 3-axis integrated accelerometers. Following appropriate skin preparation, two AgCl_2_ electrodes were placed on the muscle bellies according to the SENIAM guidelines on both the right and left side of the body [17,18,19,20]. Table 1 lists the muscles we have included in our study.

The simultaneous EMG signals, accelerometer data, and time-synchronized video signals [21,22] were recorded and stored using the EMG and Motion Tools software (version 8.11, Cometa Italy). The sampling rates for the sEMG, accelerometer data, and the video sequences were 2000, 142 Hz, and 60 frames per second, respectively.

For subsequent off-line analysis, EMG and accelerometer data were exported to txt format to be processed by an in-house written Python script (Python v. 3.12.2). Codes are available from the corresponding author, upon reasonable request.

### 2.1. Movement Execution Protocol

First, the test subject was instrumented with the sensor units and familiarized with the devices. Then the subject was instructed to perform 20 reverse punches with the dominant side (right) with approximately 5 s intervals separating each punch. The punches were delivered to a 35 × 35 cm (13.8 × 13.8 inch) semi-rigid X-ray film hung on a holder frame to maintain constant distance for the attack and to provide a target for the subject to focus on. All punches were executed correctly according to the expert (L.Cs.) supervising the execution. Prior to data collection, a warmup sequence was performed.

### 2.2. Data Processing and Evaluation

For muscle activation sequence determination, the best 5 out of 20 punches were selected. The best punches were chosen based on the maximum acceleration magnitude (in multiples of g) of the punching forearm, resulting in the greatest velocity at impact with the target. Forearm acceleration magnitudes were calculated as:

(1) $$\mathrm{accMag}=\sqrt{{{\mathrm{acc}}_{x}}^{2}+{{\mathrm{acc}}_{y}}^{2}+{{\mathrm{acc}}_{z}}^{2}},$$

where acc_x_, acc_y,_ and acc_z_, refer to acceleration vectors in x, y, and z directions.

**Figure 2:**
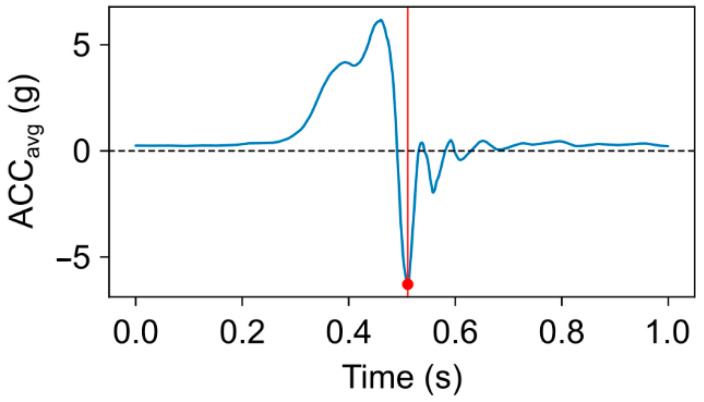
Average acceleration of the punching forearm. The end of the punch is defined at the timestamp corresponding to signal minimum (red dot), where the highest negative acceleration was measured.

For each muscle a representative EMG signal was created by signal “averaging” 0.5 s-long EMG segments taken from the 5 best punches. This procedure was carried out by aligning the ends of the EMG segments with the end-of-the-punch time index. The time index of the end-of-punch was determined as the timestamp corresponding to the local minimum of the averaged (acc_x_, acc_y_, acc_z_) forearm acceleration signal see Figure 2. Finally, the representative EMG is calculated by taking the median of the 5 EMG segments. All subsequent calculations were carried out on the “averaged” signals.

The median signal was filtered with a digital 6th order Butterworth bandpass filter between 30 and 300 Hz [23]. Since our wireless EMG system ran on USB power, we did not apply any narrow-band power-line signal filtering.

The filtered and windowed EMG was transformed with a Teager–Kaiser energy operator (TKEO) [24] to improve signal-to-noise ratio. The TKEO transformation is a method of sEMG signal processing recommended for onset detection problems dealing with rapid, explosive movements [25]. The transformation is performed according to:

(2) $$\Psi \left(x_{n}\right)=x_{n}^{2}-\left(x_{n+1}{\cdot x}_{n-1}\right),$$

where x_n_ is the nth sample of the filtered EMG signal. To acquire a smoother envelope, the transformed signal was filtered with a 4th order Butterworth low pass filter, with a 7 Hz cutoff frequency.

To visualize the muscle activation sequence pattern, we have implemented a unique technique. Instead of only characterizing muscle activation as active/non-active from the EMG data we have developed an interval representation, where using the processed EMG signal 0–25%, 25–50%, 50–75%, and 75–100%, activation levels were determined and separated as the function of time. Consequently, a more precise visualization and a better understanding of the muscle behavior and activation pattern is achievable.

**Figure 3:**
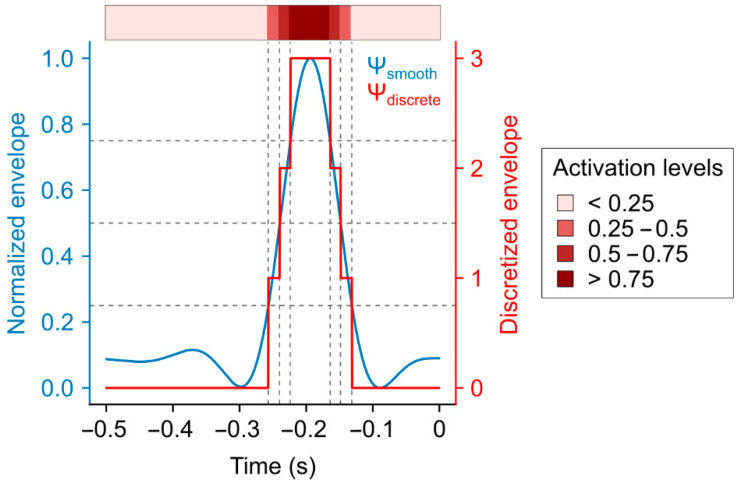
The normalized and smoothed Teager–Kaiser envelope (Ψ_smooth_) is discretized into quartiles to gain the stepped discrete envelope (Ψ_discrete_). Activation thresholds corresponding to 25%, 50%, and 75% of the normalized envelope amplitude are denoted by horizontal dashed lines, with their respective temporal occurrences marked by vertical dashed lines. The activation map on the top of the figure is constructed from Ψ_discrete_. The colors of the map indicate the level of activation, where darker colors represent greater activation. Activation onset is defined when the discretized envelope takes value 1 (activation level surpasses the value of 0.25).

The smoothed envelope was normalized to a range 0–1, then discrete values of 0, 1, 2, and 3 were assigned to the continuous amplitude values to gain activation levels.

(3) $$\Psi_{D}=\left\{\begin{array}{l} 0,\;if\;\Psi_{smooth}<0.25\\ 1,\;if\;\Psi_{smooth}\;\in [0.25,\;0.5)\\ 2,\;if\;\Psi_{smooth}\;\in [0.5,\;0.75)\\ 3,\;if\;\Psi_{smooth}\;\geq 0.75 \end{array},\right.$$

where Ψ_D_ is the discretized envelope (activation level) and Ψ_smooth_ is the low pass filtered smoothed envelope, as seen in Figure 3.

Muscle activation onset timestamp is defined as when Ψ_D_ value passed from 0 to 1 (when the normalized amplitude first surpassed the value of 0.25). These onset timestamps for each of the examined muscles are listed in Table 2.

Evaluation of the results. The performance was rated based on the recorded and processed data by an expert (8th dan), who is a practicing coach in karate. By analyzing the data, the expert determined the correct and incorrect movement execution components in the discussion section.

## 3. Results

Forearm accelerations of the best five punches in ascending order were: 25.14 g, 25.20 g, 25.48 g, 25.52 g, and 25.79 g (mean: 25.43 g, SD: 0.26 g).

**Figure 4:**
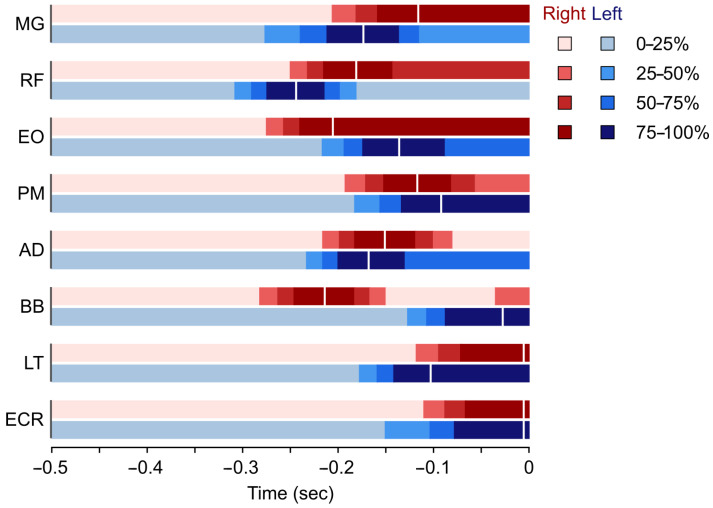
Averaged, half-second-long records of muscle activations of the arm and leg/trunk muscles during a right-hand (dominant side) Gyaku Tsuki prior to the termination of the punch/hitting the target. Different color tones represent different muscle activation levels. AD: anterior deltoid muscle, BB: biceps brachii muscle, LT: lateral head of the triceps brachii muscle, ECR: extensor carpi radialis muscle, MG: medial head of the gastrocnemius muscle, RF: rectus femoris muscle, EO: external oblique abdominal muscle, PM: pectoralis major muscle. Vertical white lines indicate the timing of the maximal activation.

Activation levels for all examined muscles were determined bilaterally. Results are displayed in Figure 4 as a visual summary of Table 2, which lists the sequence of muscle activation and activation times related to the end-of-punch.

## 4. Discussion

This study was designed to develop a method to describe the kinetic and kinematic characteristics of the traditional karate Gyaku Tsuki strike. Our aim was to determine the activation sequence of the lower and upper extremities along with the muscles of the trunk throughout the execution of the technique. Although each of the applied methods are well-documented in the literature, the novelty of this paper lies within the unique combination of the above-mentioned techniques.

The study revealed that the athlete first used the rectus femoris muscle of the front leg to stabilize the stance. From the coach’s point of view, this is the correct execution, since leg strength is strongly associated with peak punch force within elite boxers [26] and effective punches require the knee of the front leg to not move backwards, as this causes a significant reduction in efficiently transferring ground reaction forces to the punch.

Next, the right biceps brachii muscle, followed by the left (front) medial gastrocnemius almost simultaneously (0.002 s difference) with the right extensor oblique abdominal muscle, become active. Minimal stretching of the biceps brachii muscle is necessary during the initiation of the punch because the weight of the punching arm starting from the side of the torso must be maintained. Activation and contraction of the left medial gastrocnemius facilitates forward translation of the knee joint (in the direction of the attack), thus preventing backward movement of the joint. The right Abdominal External Oblique muscle plays a significant role in the rotation of the pelvis and trunk, which is supported by the next tensing muscle—the rectus femoris—on the dominant side (rear leg). The rectus femoris contraction assists in the conversion of the vertical ground reaction force component into a horizontally directed force.

The left anterior deltoid and left external oblique abdominal muscles began to be activated almost simultaneously (Δt = 0.001 s) to initiate withdrawal of the extended left arm and to help accelerate the punch performed with the right.

Rotation of the right upper arm in the sagittal plane (initiated earlier by the biceps brachii muscle) is further accelerated by the right anterior deltoid muscle, which was involved in the next movement sequence.

Next, the medial gastrocnemius muscle of the back leg was activated. By analyzing the data, the expert concluded that the right medial gastrocnemius muscle should have engaged much earlier, at about the same time as the rectus femoris of the front leg (left side). Based on this, it allows us to conclude that the subject could not efficiently harness the ground reaction force but tried to fix the back foot by pressing the heel to the ground instead of plantarflexion. This is also clearly visible on the time-synchronized video footage, where the inner sole of the rear foot is pressed against the ground, see Figure 1C. Based on these observations, our recommendation for coaches is that through strengthening lower limb muscles involved in the movement, they can improve stance stability, which results in higher punch impact force.

The right and the left major pectoral muscles are activated next, respectively, with a negligible 0.01 s difference between the sides. While the right pectoral muscle directed the punching arm towards the target positioned at the midline of the body, the left muscle brought the retracting opposite arm closer to the trunk.

Closing to the end of the movement, muscles of the left arm (lateral triceps, extensor carpi radialis and biceps brachii muscles, in order) activated before their counterparts on the right side. Finally, the focus of punch is ensured by fully extending the right elbow joint by the right lateral triceps [27] and stabilizing the right wrist with the assistance of the right extensor carpi radialis muscle.

Overall, it can be said that the pelvis/trunk rotation is the most decisive accelerating element in the entire process. Considering that the whole execution time takes ~0.3 s, it can be said that the karate Gyaku Tsuki movement pattern is the result of very fast, almost simultaneous contraction of the 16 examined muscles.

### Future Development Possibilities

To generate a universally useful database, the recording of a large data pool is necessary, and, by using AI machine learning tools, the determination of optimal execution would be possible. Consequently, by pairing the subjective rating of the execution by the experts and the recorded data automated evaluation of the punch can be achieved. To develop and train supervised machine learning models, an expert consensus-based feature set on a pre-defined ordinary scale should be developed, which clearly distinguishes correct/incorrect punch executions.

It is worth mentioning that this method can be applied to superficial muscle components of other kinetic chains in the body and can be generalized to analyze other movement patterns in other sports.

## 5. Conclusions

By creating unique and simple data recordings, data evaluation, and representation methods for muscle activation, the analysis of the Gyaku Tsuki karate punch is possible. The method provides data that is not only useful for the biomechanics expert but also for the coaches working with the athletes. By implementing the method introduced in the paper determination of faulty execution and identification of correct and incorrect movement patterns is possible.

A novel method for muscle activation analysis gives objective additional information for “coaches eye”, which helps to develop karate techniques through evaluation of the execution, meaning “good form” according the World Karate Federation (WKF) competition rules: “Good form is the properly executed technique which have the characteristics conferring probable effectiveness within the framework of traditional karate concept.” [28] Using the method of our study, either for the coaches or for the athletes, it is possible to motor control the execution by knowing the muscle sequences, in order to reach the highest velocity with minimum effort from the muscles. In addition, it helps to prevent injuries during training.

## 6. Limitations

Despite the fact that the aim of this study was to introduce our proposed method, the obvious limitation is that only one subject participated in data recording.

## Figures and Tables

**Table 1:** EMG/Accelerometer sensor unit positioning with abbreviations. Based on the SENIAM EMG placement protocol.

Electrode	Side	Muscle (Abbreviation)	Function
1	Right	Gastrocnemius, medial head (MG)	Ankle plantar flexion
2	Left		
3	Right	Rectus femoris (RF)	Knee extension
4	Left		
5	Right	Abdominal External Oblique (EO)	Trunk rotation
6	Left		
7	Right	Major Pectoral (PM)	Arm adduction
8	Left		
9	Right	Deltoid, anterior head (AD)	Shoulder anteflexion
10	Left		
11	Right	Biceps Brachii (BB)	Elbow stabilization
12	Left		
13	Right	Triceps Brachii, lateral head (LT)	Elbow extension/stabilization
14	Left		
15	Right	Extensor Carpi Radialis (ECR)	Wrist stabilization
16	Left		

**Table 2:** Muscle activation times of the arm and leg/trunk muscles during a right-handed (dominant side) Gyaku Tsuki. Negative activation times refer to durations before the termination of the punch (0 s).

Sequence	Side	Muscle	Activation Time (s)
1	Left	Rectus Femoris	−0.308
2	Right	Biceps Brachii	−0.282
3	Left	Medial Gastrocnemius	−0.277
4	Right	Abdominal External Oblique	−0.275
5	Right	Rectus Femoris	−0.250
6	Left	Anterior Deltoid	−0.233
7	Left	Abdominal External Oblique	−0.217
8	Right	Anterior Deltoid	−0.216
9	Right	Medial Gastrocnemius	−0.206
10	Right	Major Pectoral	−0.193
11	Left	Major Pectoral	−0.183
12	Left	Lateral Triceps	−0.178
13	Left	Extensor Carpi Radialis	−0.151
14	Left	Biceps Brachii	−0.127
15	Right	Lateral Triceps	−0.118
16	Right	Extensor Carpi Radialis	−0.110

## Data Availability

Raw EMG and accelerometer data is available to download as the Supplementary S1.

## Supplementary Materials

The following supporting information can be downloaded at: https://www.mdpi.com/article/10.3390/sports13070218/s1, Data supplement S1: Raw EMG and Accelerometer data file.

## Abbreviations

The following abbreviations are used in this manuscript:

ACC	Acceleration
AD	Deltoid muscle, anterior head
BB	Biceps Brachii muscle
ECR	Extensor Carpi Radialis muscle
EO	Abdominal External Oblique muscle
LT	Triceps Brachii, lateral head
MG	Gastrocnemius, medial head
PM	Major Pectoral muscle
RF	Rectus Femoris muscle
SD	Standard Deviation
sEMG	Surface ElectroMyoGraphy
SENIAM	Surface ElectroMyoGraphy for the Non-Invasive Assessment of Muscles
TKEO	Teager–Kaiser Energy Operator
WKF	World Karate Federation
